# Chiral Amino Acids Mediate Mitochondria-Dependent Apoptosis of Human Proximal Tubular Epithelial Cells Under Oxidative Stress

**DOI:** 10.3390/ijms252413439

**Published:** 2024-12-15

**Authors:** Ying Lu, Yang Zhang, Zhaoyang Jin, Shuaishuai Cui, Li Wu, Yujian He

**Affiliations:** 1 School of Chemical Sciences, University of Chinese Academy of Sciences, Beijing 100049, China; luying21@mails.ucas.ac.cn (Y.L.); jinzhaoyang20@mails.ucas.ac.cn (Z.J.); cuishuaishuai22@mails.ucas.ac.cn (S.C.); 2 School of Future Technology, University of Chinese Academy of Sciences, Beijing 100049, China; zhangyang184@mails.ucas.ac.cn

**Keywords:** chiral amino acids, HK-2 cells, ROS, oxidative stress, mitochondria-dependent, apoptosis

## Abstract

Amino acids are the basic structural units of life, and their intake levels affect disease and health. In the case of renal disease, alterations in amino acid metabolism can be used not only as a clinical indicator of renal disease but also as a therapeutic strategy. However, the biological roles and molecular mechanisms of natural chiral amino acids in human proximal tubular epithelial cells (HK-2) remain unclear. In this study, cell viability assays revealed that chiral acidic amino acids (Glu and Asp) and aromatic amino acids (Trp and Phe) inhibited cell growth. The molecular mechanisms indicated that cell growth was closely related to ROS levels. Specifically, chiral Glu, Asp, Trp, and Phe induced oxidative stress and mitochondria-dependent apoptosis in HK-2 cells. This was manifested by elevated levels of intracellular ROS, 8-OHdG, and MDA, increased activities of antioxidant enzymes CAT, SOD, and GPx, decreased mitochondrial membrane potential, increased cytoplasmic Ca^2+^ concentration, and cell acidification. The expression levels of apoptosis-related molecules Caspase-9, Caspase-3, Cyt-C, and Bax were increased, and the expression level of anti-apoptotic molecule Bcl-2 was decreased. Moreover, L-Glu, D-Asp, L-Trp, and D-Phe exhibited a more pronounced inhibition of cell growth and elicited more substantial alterations in gene expression compared to the other configurations.

## 1. Introduction

Amino acids are the basic building blocks of biologically functional macromolecular proteins [1]. There are 20 amino acids involved in protein synthesis, categorized as neutral, acidic, and basic [2]. In addition, amino acids (except glycine) are also chiral molecules, classified into L-configuration and D-configuration amino acids (L/D-AAs) [3]. Among them, the most nutritionally valuable amino acids exist as the L-isomers, and natural proteins are entirely composed of L-AAs [4]. Indeed, many foods contain considerable amounts of D-AAs: we consume more than 100 mg of D-AAs every day. Interestingly, D-AAs are formed during food processing and originate from microorganisms, water, soil, and other environments [5]. Dairy products have been reported to contain amounts of D-Ala, D-Asp, and D-Glu (1–3 mg/L) [6]. Recent technical developments in chiral amino acid metabolomics made it possible to distinguish between L-AAs and D-AAs, revealing the existence of D-AAs in the living world and suggesting new directions for studying cell biology based on chiral amino acids [7].

Amino acids serve as structural elements and energy sources required for normal growth, differentiation, and function of cells [8]. Different cell types, cellular metabolic states, and microenvironments have various demands for chiral amino acids [9]. Therefore, the intake of chiral amino acids directly affects cellular physiological functions, subsequently impacting the health of the organism. Studies have shown that dietary restriction of L-methionine can extend the lifespan of mice and rats [10,11,12]. AXA1125, an oral endogenous regulator containing five L-AAs (leucine, isoleucine, valine, arginine, and glutamine) and an amino acid precursor (N-acetylcysteine) in specific proportions, has been shown in clinical studies to treat non-alcoholic fatty liver disease. AXA1125 exhibits higher bioactivity compared to placebo, with a greater reduction in patient inflammation markers [13,14,15]. Additionally, D-serine holds great potential in early diagnosis, monitoring, and prognosis of renal function and chronic kidney disease [16]. However, the accumulation of amino acids in certain tissues can also cause serious damage. For example, injecting rats with D-serine results in growth inhibition [17]. Thus, the biological functions of different amino acids and their roles in pathophysiology vary and remain incompletely understood.

In mammals, amino acid metabolism primarily utilizes amino acid oxidases. Amino acid oxidases include L-amino acid oxidase (LAAO), D-amino acid oxidase (DAAO), and specific amino acid oxidases, which are predominantly located in the peroxisomes of proximal renal tubular epithelial cells. Furthermore, human proximal renal tubular epithelial cells (HK-2) are widely used as an in vitro model for evaluating drug nephrotoxicity due to their morphological and functional similarities with primary human proximal renal tubule epithelial cells [18,19,20]. Amino acids are oxidatively deaminated by amino acid oxidases to produce α-keto acids, NH_3_, and hydrogen peroxide (H_2_O_2_) [21,22]. H_2_O_2_, a form of reactive oxygen species (ROS), is toxic to most cells. To maintain cellular redox homeostasis, organisms have evolved effective antioxidant defense systems, in which the enzymatic antioxidant system is mainly composed of superoxide dismutase (SOD), catalase (CAT), glutathione peroxidase (GPx), and glutathione reductase (GR) [23]. However, if the excess ROS is not effectively eliminated, cells can be destroyed by oxidative stress, thereby inducing disease [24]. D-serine-induced nephrotoxicity has been reported to correlate with ROS production in response to DAAO [25]. In addition, excess ROS can also directly attack the mitochondrial membrane, causing changes in mitochondrial membrane permeability, decreasing mitochondrial membrane potential (MMP), and releasing apoptotic factors, which in turn activate the cysteinyl aspartate specific proteinase (Caspase) cascade signaling, ultimately leading to apoptosis [26,27]. To date, the correlation and molecular pathway mechanism linking chiral amino acids to oxidative damage in HK-2 cells has not been thoroughly investigated. In this study, we constructed an in vitro model of 18 chiral natural amino acids acting on HK-2 cells. We assessed cell viability from the perspective of species and chirality, examined their association with oxidative stress, and elucidated the molecular mechanism underlying apoptosis through mitochondrial dysfunction. This study aims to offer valuable insights for disease prevention and the development of amino acid-based drugs.

## 2. Results

### 2.1. Effect of Chiral Amino Acids on the Viability of HK-2 Cells

Since tyrosine is insoluble and glycine is achiral, the remaining 18 natural chiral amino acids (2.5–30 mM) were chosen to treat HK-2 cells for 24 h and categorized according to acidic, basic, neutral, and aromatic amino acids for comparison. CCK-8 was used to determine the viability of HK-2 cells at different doses.

**Figure 1:**
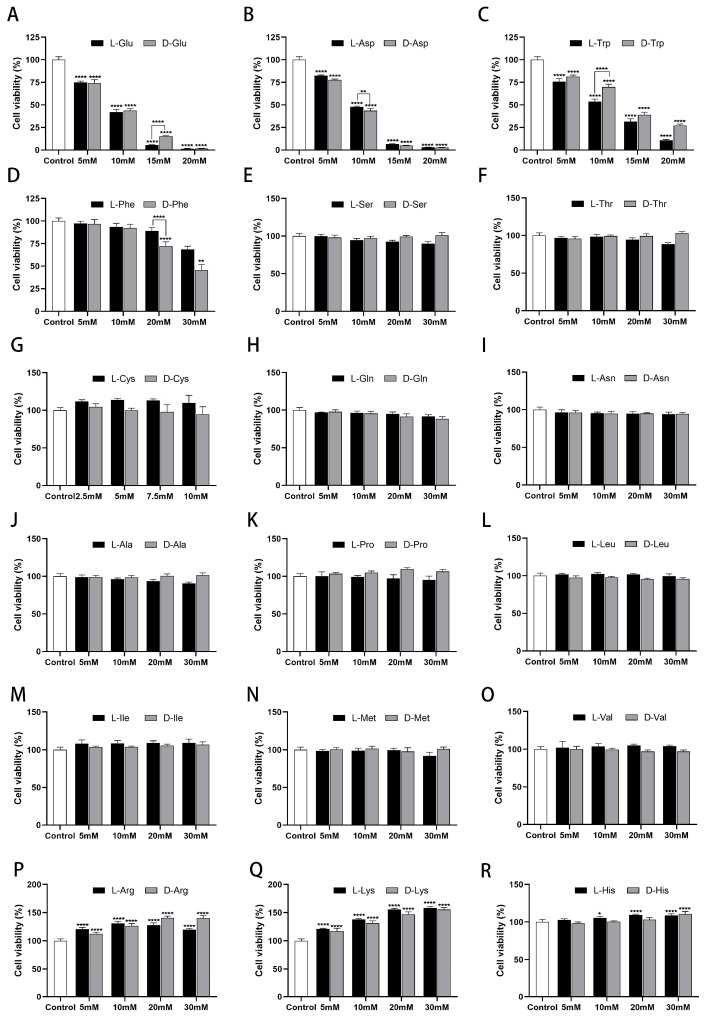
Effects of chiral amino acids on HK-2 cells’ viability. (**A**,**B**) Acidic amino acid (Glu, Asp). (**C**,**D**) Aromatic amino acids (Trp, Phe). (**E**–**O**) Neutral amino acids. (**P**–**R**) Basic amino acids. (Statistical significance relative to control was marked with * *p* < 0.05, ** *p* < 0.01, or **** *p* < 0.0001).

Cell viability showed a dose-dependent (5–30 mM) decrease after exposure to acidic and aromatic amino acids (Glu, Asp, Trp, and Phe) (Figure 1A–D). Of these, Glu, Asp, and Trp showed remarkable inhibition, and Phe showed slight inhibition. Differently, cell viability showed a dose-dependent (5–30 mM) increase after exposure to basic amino acids (Arg, Lys, and His) (Figure 1P–R). Thereinto, Arg and Lys have a significant effect and will promote cell growth by 1.3–1.5 times; His has a weak effect and will promote cell growth by 1.1 times. Cell viability was not greatly altered after exposure to the remaining 11 amino acids (2.5–30 mM) for 24 h, which all remained above 95% (Figure 1E–O). From a chiral perspective, L-Glu, D-Asp, L-Trp, and D-Phe demonstrated more potent inhibition of cell growth compared to the other configurations. Specifically, L-Trp (10 mM) resulted in 16.28% lower cell viability than D-Trp. Furthermore, when the concentration of L/D-Glu, Asp, and Trp was 10 mM, the cell viability was near 50%, which closely aligns with the previously reported IC50 (L-Glu) values of 13 mM [28] and 15 mM [29]. Therefore, the 10 mM concentration of chiral amino acids was initially selected for subsequent experiments.

### 2.2. Effects of Chiral Amino Acids on Oxidative Stress of HK-2 Cells

**Figure 2:**
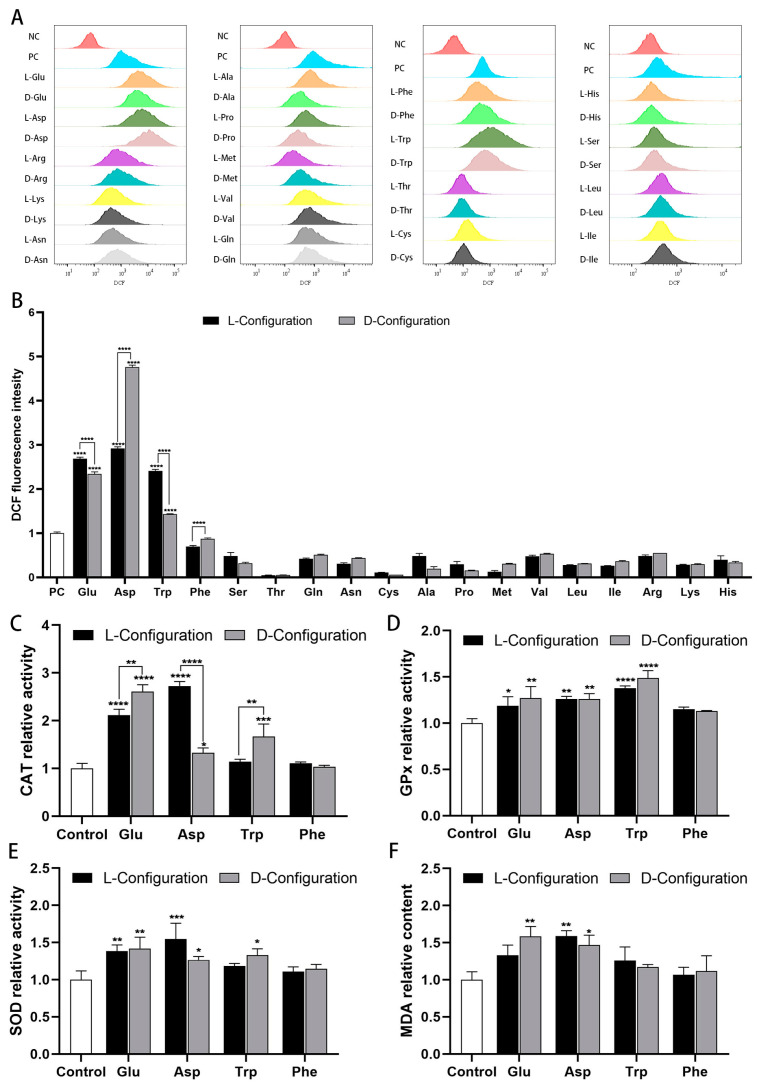
Oxidative stress induced by 10 mM of chiral amino acids acting on HK-2 cells for 24 h. (**A**) Fluorescence distribution of DCFH-DA probe, where negative control (NC) was without amino acids and ROS-up treatment, and positive control (PC) was with ROS-up treatment. (**B**) Mean fluorescence intensity of the DCFH-DA probe, where all groups were subtracted from the autofluorescence value of the negative control. Relative activity of (**C**) catalase, (**D**) glutathione peroxidase, (**E**) superoxide dismutase, and (**F**) lipid peroxidation. (Statistical significance relative to control was marked with * *p* < 0.05, ** *p* < 0.01, *** *p* < 0.001, or **** *p* < 0.0001).

To evaluate the effects of chiral amino acids on oxidative stress in HK-2 cells, a 2′,7′-dichlorodihydrofluorescein diacetate (DCFH-DA) fluorescent probe was used to detect the intracellular ROS level. Concurrently, we detected CAT, SOD, GPx activities (antioxidant indicators), malondialdehyde (MDA) and 8-hydroxy-2′-deoxyguanosine (8-OHdG) content (oxidative indicators) in HK-2 cells. The results revealed significant differences in the distribution of cellular fluorescence under the effect of different amino acids. Notably, acidic and aromatic amino acids (Glu, Asp, Trp, and Phe) that inhibited cell growth induced an extreme increase in cellular fluorescence intensity, indicating a significant rise in intracellular ROS levels (Figure 2A). Specifically, the mean fluorescence intensity of cells exposed to chiral Glu, Asp, and Trp (10 mM) escalated by a factor of 1–5 compared to the positive control group. The mean fluorescence intensity of cells exposed to chiral Phe (10 mM) was marginally lower than that of the positive control, but still significantly higher than that of the other treatment groups (Figure 2B). Moreover, compared to the control group, chiral Glu, Asp, and Trp notably enhanced the activities of CAT, SOD, and GPx activities to catabolize H_2_O_2_ and catalyze the oxidation of reduced glutathione (Figure 2C–E). Meanwhile, there was a notable increase in the concentration of 8-OHdG (Figure S1) and a rise in the relative content of MDA (Figure 2F). Chiral Phe slightly activated antioxidant enzyme activities, 8-OHdG, and MDA content. Regarding chirality, D-Glu, L-Asp, and D-Trp activated CAT to a greater extent than the other configurations, resulting in less ROS, which corresponded to their weaker toxicity on HK-2 cells.

### 2.3. Effect of Chiral Amino Acid on the Morphology and Apoptosis Rate of HK-2 Cells

**Figure 3:**
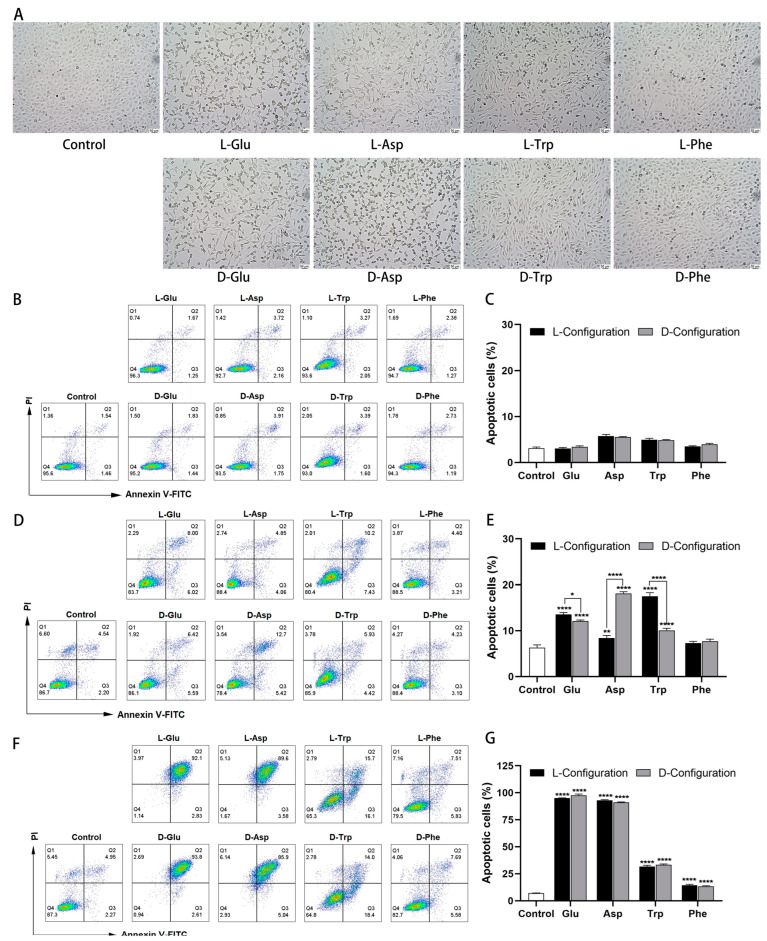
Morphological changes and apoptosis induced by chiral amino acids in HK-2 cells under oxidative stress. (**A**) Cell morphology after 15 mM of chiral amino acids acting on HK-2 cells for 24 h, observed with an inverted microscope at a magnification of 10×. Scale bars: 50 µm. Double staining of apoptotic cells and quantification of apoptosis rate after (**B**,**C**) 10 mM, (**D**,**E**) 15 mM, and (**F**,**G**) 20 mM of chiral amino acids acting on HK-2 cells for 24 h. (Statistical significance relative to control was marked with * *p* < 0.05, ** *p* < 0.01, or **** *p* < 0.0001).

After the HK-2 cells were treated with the chiral amino acid for 24 h, the cell morphology was observed by an inverted microscope. The results showed that control cells were full, short spindle-shaped, and showed tightly adherent growth. When the concentration of chiral Glu, Asp, Trp, and Phe was 10 mM, the HK-2 cell gap increased, and the cell density decreased (Figure S2). Further increasing the concentration to 15 mM, the cells thinned to a thin strip, the cell space enlarged considerably, the adhesion worsened, and some cells floated (Figure 3A). In addition, the cells at 20 mM were close to death with pyknotic and fragmented nuclei (Figure S3).

To assess the effect of chiral amino acids in inducing apoptosis of HK-2 cells under oxidative stress, the fluorescence intensity of Annexin V-FITC/PI was quantified using flow cytometry. When exposed to 10 mM chiral Glu, Asp, Trp, and Phe, the percentage of total apoptotic cells (including early and late apoptosis) ranged from 3.07 to 5.73%, which was close to the control (3%) (Figure 3B,C), indicating that this concentration induced only weak apoptosis. The apoptosis rate increased to 7.25–18.15% at a concentration of 15 mM, significantly enhanced compared to the control (6.32%) (Figure 3D,E), indicating that the cells partially significant early apoptosis. The apoptosis rate was close to the data reported in the literature, with an increase of 22.46% [28] and 6.34% [29] after the action of 13 mM and 15 mM L-Glu on PC12 cells, respectively. Moreover, L-Glu, D-Asp, and L-Trp elicited more pronounced apoptosis compared to the other configurations, aligning with their heightened cytotoxicity. Specifically, the apoptosis rate of D-Asp (15 mM) was 9.76% higher than that of L-Asp, and L-Trp exhibited a 7.46% higher rate than D-Trp. Upon further increasing the concentration to 20 mM, 91.12–97.61% of cells in the Glu and Asp treatment groups died in the form of late apoptosis, while 14.31% and 33.26% of cells in the Phe and Trp treatment groups underwent apoptosis, respectively (Figure 3E,F). For a comprehensive comparison, chiral amino acids at a concentration of 15 mM were selected for subsequent studies.

### 2.4. Effects of Chiral Amino Acids on Mitochondrial Function of HK-2 Cells

**Figure 4:**
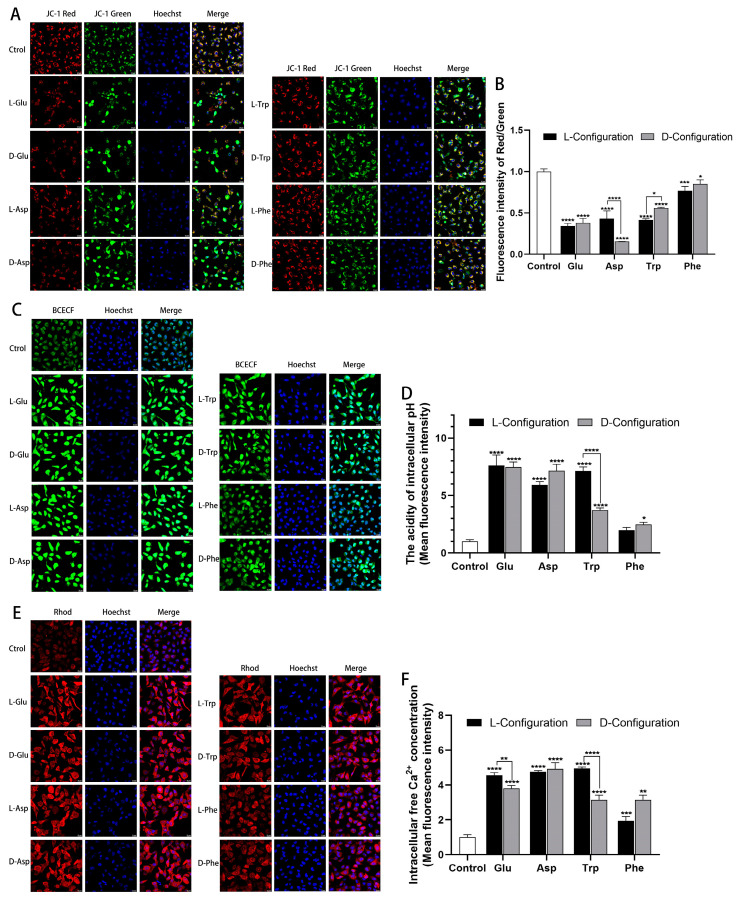
Mitochondrial dysfunction induced after 15 mM of chiral amino acids acting on HK-2 cells for 24 h. (**A**) Representative confocal images of HK-2 cells stained with JC-1 (red and green) and Hoechst 33342 (blue). Scale bars: 20 µm. (**B**) Quantification of the level of MMP. (**C**) Representative confocal images of HK-2 cells stained with BCECF (green) and Hoechst 33342 (blue). Scale bars: 20 µm. (**D**) Quantification of the extent of cell acidification. (**E**) Representative confocal images of HK-2 cells stained with Rhod (red) and Hoechst 33342 (blue). Scale bars: 20 µm. (**F**) Quantification of the concentration of intracellular free Ca^2+^. (Statistical significance relative to control was marked with * *p* < 0.05, ** *p* < 0.01, *** *p* < 0.001, or **** *p* < 0.0001).

To investigate the alteration of cellular mitochondrial function after exposure to chiral amino acids, a JC-1 probe, BCECF probe, and Rhod probe were used to detect cellular MMP, pH, and Ca^2+^ levels, respectively. Compared with the control group, after exposure to chiral Glu, Asp, Trp, and Phe, the red fluorescence intensity of the JC-1 probe in cells was significantly weakened, and the green fluorescence intensity was significantly enhanced (Figure 4A). The red/green fluorescence intensity of the JC-1 probe was notably reduced, indicating a decrease in MMP in cells (Figure 4B). In terms of chirality, the red/green fluorescence intensity elicited by D-Asp and L-Trp (15 mM) was lower than the other configurations by 27% and 13%, respectively. This indicated that D-Asp and L-Trp induced a more pronounced reduction in MMP, correlating with a greater apoptosis rate. In addition, the green fluorescence intensity of the BCECF probe was profoundly amplified after exposure to four chiral amino acids, indicating that the intracellular pH was obviously decreased and cell acidification was aggravated (Figure 4C). Among them, L-Trp (15 mM) induced 340% higher green fluorescence intensity than D-Trp, causing more visible cell acidification, corresponding to more cell apoptosis (Figure 4D). Similarly, the red fluorescence intensity of the Rhod probe treated with chiral amino acid groups was notably increased, suggesting a substantial increase in intracellular free Ca^2+^ concentration and the release of Ca^2+^ from mitochondria to the cytoplasm (Figure 4E). Red fluorescence intensity induced by L-Glu and L-Trp (15 mM) was 76% and 180% higher than that of the other configurations, respectively, representing that they induced more Ca^2+^ release, corresponding to more cell apoptosis (Figure 4F).

### 2.5. Effect of Chiral Amino Acids on the Expression of Mitochondria-Dependent Apoptosis-Related Proteins of HK-2 Cells

**Figure 5:**
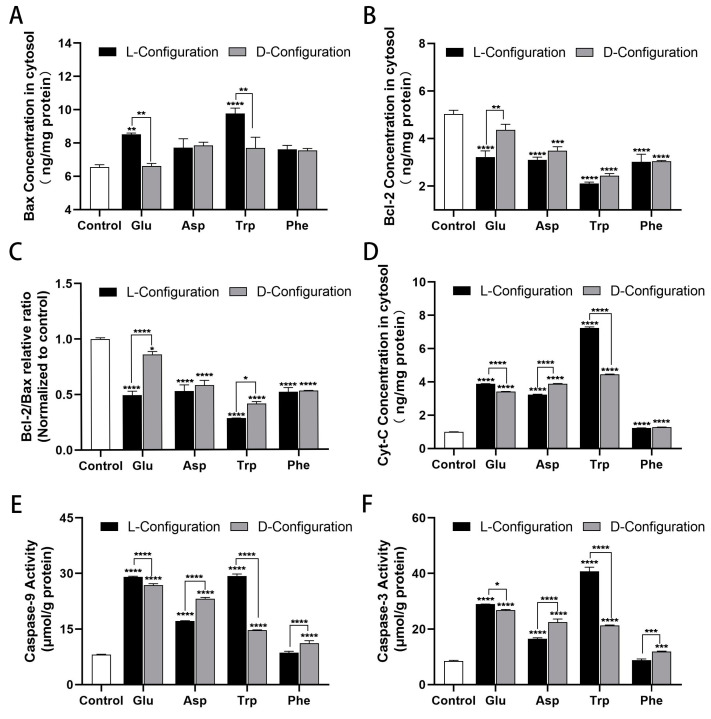
Variable expressions of mitochondria-dependent apoptosis-related proteins induced after 15 mM of chiral amino acids acting on HK-2 cells for 24 h. (**A**,**B**) Bax and Bcl-2 protein concentrations. (**C**) Relative ratio of Bcl-2/Bax protein after normalization. (**D**) Cyt-C protein content. (**E**,**F**) Caspase-9 and Caspase-3 activity. (Statistical significance relative to control was marked with * *p* < 0.05, ** *p* < 0.01, *** *p* < 0.001, or **** *p* < 0.0001).

To investigate the mechanism of mitochondria-dependent apoptosis induced by chiral amino acids in HK-2 cells, the expression of relevant proteins was examined using ELISA. The results showed that the content of the cellular anti-apoptotic protein b-cell lymphoma-2 (Bcl-2) was obviously decreased, the content of the pro-apoptotic protein Bcl-2 associated X protein (Bax) was notably increased, and the relative ratio of Bcl-2/Bax was significantly decreased after treatment with 15 mM of chiral Glu, Asp, Trp, and Phe (Figure 5A–C). Particularly, L-Glu and L-Trp induced more observable Bcl-2 reduction and Bax elevation relative to the other configurations, thereby promoting greater cellular apoptosis. Furthermore, the cytochrome C (Cyt-C) protein content and the activity of Caspase-9 and Caspase-3 were significantly elevated (Figure 5D–F). Especially, L-Glu, D-Asp, L-Trp, and D-Phe induced remarkably higher levels of Cyt-C, Caspase-9, and Caspase-3 proteins in HK-2 cells than the other configurations, giving rise to higher apoptosis rates.

## 3. Discussion

**Figure 6:**
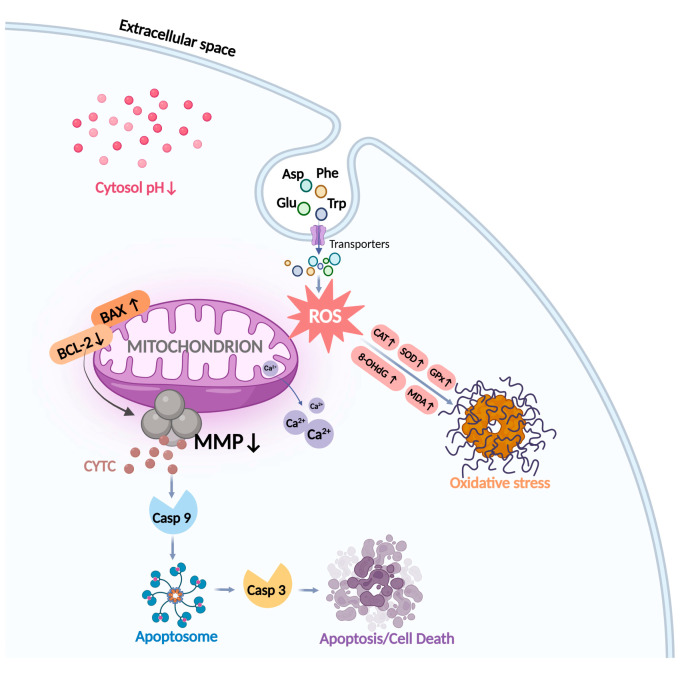
Diagram of the molecular mechanism of chiral amino acid action in HK-2 cells. After exposure to Glu, Asp, Trp, and Phe, the mitochondrial ROS levels in HK-2 cells increased, inducing oxidative damage to DNA and lipids. Redox homeostasis is regulated by elevating antioxidant enzyme levels, and an imbalance in this homeostasis leads to oxidative stress. Meanwhile, decreased cellular MMP, mitochondrial Ca^2+^ release, and cellular acidification mediated altered mitochondrial function, which further induced apoptosis through the action of the Bcl-2 family, Cyt-C, and Caspase family protein pathways.

In this study, we found that chiral acidic amino acids (Glu and Asp) and chiral aromatic amino acids (Trp and Phe) induced oxidative stress and mitochondria-dependent apoptosis in HK-2 cells and mapped a plausible molecular mechanism (Figure 6).

First, for the doses of amino acids used, the doses exposed in vivo and in cellular models are typically 1–50 mM [28,30,31,32,33]. In addition, normal adults have a dietary intake of 2–15 g/d of amino acids and a blood amino acid concentration of 70–350 μM [34]. The concentrations tested in this study were 2.5–50 mM, about 100 times greater than the normal in vivo concentrations tested. This is because the concentration of amino acids in the body increases during the onset of the disease, e.g., the plasma D-serine levels of CKD patients are 5-fold to 10-fold higher than those of the healthy population, and D-serine levels in the spinal cord of patients with amyotrophic lateral sclerosis (ALS) are also elevated [31,35]. Furthermore, owing to the short exposure period (1 day) in our in vitro study, the use of an order of magnitude higher amino acid concentration was adequate. This study found that acidic amino acids (Glu and Asp) containing carboxyl groups and aromatic amino acids (Trp and Phe) containing benzene rings exhibited a dose-dependent inhibition (5–30 mM) of HK-2 cell growth. It has been reported that 5 mM Glu also induces excitotoxicity in the mouse hippocampal neuronal HT-22 cells [36]. Similarly, in CHO and HeLa cells, Phe, Trp, Asp, and Glu (10 mM and 20 mM) can also inhibit cell proliferation [37].

ROS consist of superoxide anion (O_2_^-^), hydrogen peroxide (H_2_O_2_), hydroxyl radicals (OH^-^), ozone (O_3_), and singlet oxygen (^1^O_2_), which can cause lipid peroxidation, DNA fragmentation, and protein oxidation in multiple cell types [38]. As reported, L-Glu induces neuronal cell neurotoxicity by upregulating ROS [25,30]. Cellular senescence upregulates DAAO levels to produce ROS-induced oxidative DNA damage [39]. Oxidative stress could be induced by excess ROS production. In this paper, we quantitatively detected that the regulatory effects of amino acids on HK-2 cell growth are closely related to ROS production. Specifically, acidic (Glu, Asp) and aromatic amino acids (Trp, Phe) inhibited cell growth, induced intracellular accumulation of ROS, 8-OHdG, and MDA, and elevated levels of the antioxidant enzymes CAT, SOD, and GPx. The MDA content can reflect the degree of lipid peroxidation in the organism to some extent [40], indicating the increased lipid oxidation in HK-2 cells. 8-OHdG is a product formed after reactive oxygen free radicals oxidatively damage nuclear DNA or mitochondrial DNA [41]. The elevated concentration of 8-OHdG suggested oxidative damage to both the nuclear and mitochondrial DNA in HK-2 cells. The elevated antioxidant enzyme content indicated that the chiral amino acids activated the cellular antioxidant defense system and increased the cellular antioxidant capacity to resist oxidative damage. What is more, the main product of chiral amino acid metabolism was H_2_O_2_, which corresponded to the most significant activation of CAT activity. This is in line with the reported experimental results that chiral amino acid stimulation of Saccharomyces cerevisiae induced changes mainly in the contents of H_2_O_2_ and CAT [42].

The primary location of ROS generation is the mitochondria. Mitochondrial membrane potential (MMP) is an essential biomarker of apoptosis, and its production is induced by oxidative stress [43]. The alteration of MPP is a major mechanism of the apoptotic signaling pathway in mitochondria [44]. In addition, the mitochondria are involved in regulating intracellular pH and cytosolic Ca^2+^ [45]. Reduction of cytosolic pH may directly affect MMP to make it decrease, further leading to mitochondrial dysfunction [45]. A study showed that ROS-induced intracellular acidification and mitochondria-mediated cytosolic pH reduction were associated with mitochondria-mediated apoptosis [46]. The Ca^2+^ concentration also plays a crucial role in the regulation of apoptosis. It has been reported that ROS can stimulate the increase in intracellular free Ca^2+^ concentration, resulting in the accumulation of Ca^2+^, breaking the cell Ca^2+^ homeostasis, and triggering the apoptosis pathway [47]. In our study, chiral Glu, Asp, Trp, and Phe mediated a significant decrease in MMP, a substantial reduction in intracellular pH, and the release of mitochondrial Ca^2+^, and contributed to an increase in apoptosis in HK-2 cells. This revealed that chiral Glu, Asp, Trp, and Phe interrupted the homeostasis of mitochondria in HK-2 cells, mediated mitochondrial dysfunction in response to oxidative stress, and promoted mitochondria-dependent apoptosis.

Numerous genes and proteins can influence the progression of apoptosis along the mitochondrial pathway [48]. The most vital apoptosis-related genes are proteins of the Bcl-2 family and Caspases. The Bcl-2 family is divided into two groups: anti-apoptotic (Bcl-2 and Bcl-xL) and pro-apoptotic (Bax and Bad) proteins [49,50]. The aberrant expression levels of both pro-apoptotic and anti-apoptotic proteins are believed to dictate cell death or survival by modulating apoptosis [51]. This study found that chiral Glu, Asp, Trp, and Phe increased the expression of the cellular pro-apoptotic protein BAX, decreased the expression of the anti-apoptotic protein Bcl-2, and significantly reduced the relative ratio of Bcl-2/Bax, which in turn formed a pro-apoptotic mechanism. Caspases are the executioners of apoptosis [52]. Oxidative stress can lead to the release of Cyt-C and Caspase activation [53,54]. The released Cyt-C combined with apoptotic protease activating factor-1 (Apaf-1) to trigger conformational changes, so that Caspase-9 was recruited into the apoptotic body complex [55]. The apoptotic body assembled by the recombinant component was in a wheel shape, and about seven Apaf-1 molecules and Caspase-9 formed a dimer complex structure. The allosteric activation of Caspase-9 in the apoptosome leads to the proteolysis and activation of Caspase-3, the downstream executor, which then cleaves hundreds of substrates, some of which are responsible for cell apoptosis [56]. In this study, chiral Glu, Asp, Trp, and Phe were found to significantly increase the expression levels of Cyt-C, Caspase-9, and Caspase-3. This hinted that chiral amino acid-induced oxidative stress altered MMP and promoted the release of Cyt-C from mitochondria into the cytoplasm, which further induced HK-2 apoptosis via the Caspase pathway, the occurrence of which was highly correlated with mitochondria.

Chirality is a fundamental characteristic of life processes. This study suggested that different configurations of amino acids elicited varying degrees of expression in oxidative stress indicators and mitochondria-dependent apoptosis genes in HK-2 cells. Specifically, L-Glu, D-Asp, L-Trp, and D-Phe induced more pronounced changes in ROS, antioxidant enzymes, MMP, and apoptosis-related proteins compared to the other configurations. Consequently, they exerted a more potent inhibitory effect on cell growth and induced a greater degree of cell apoptosis. This is consistent with the results reported in the literature, specifically that 10 mM L-Glu and D-Asp exert a more potent inhibitory effect on cell growth than the other configurations in CHO and HeLa cells [37]. This indicates that amino acids of different types and configurations have distinct impacts on cellular functions, revealing the diverse roles of chiral amino acids in the orchestration of life processes.

This study has the following limitations. We are interested in the drug development of combined amino acids, but the effects of different combinations of amino acids on renal cells are unclear [8]. In addition, the in vivo effects of chiral amino acids on different species still need to be confirmed [32,57]. Notably, there are apparent amino acid metabolic abnormalities in the serum of clinical renal cancer patients. When utilizing serum amino acids as clinical markers, it is imperative to take into account the influence of individual variations and other factors on the outcomes [58,59].

## 4. Materials and Methods

### 4.1. Reagents

Chiral natural amino acids were purchased from J&K Scientific Co., Ltd. (Beijing, China). Dulbecco's Modified Eagle Medium/Nutrient Mixture F-12 (DMEM-F12) and Dulbecco's Phosphate-Buffered Saline (DPBS) were purchased from Thermo Fisher Scientific Co., Ltd. (Waltham, MA, USA). The 6-, 12-, and 96-well cell culture plates and confocal glass well plates, 60 mm and 100 mm cell culture dishes, and cell spatulas were purchased from Corning Co., Ltd. (Corning, NY, USA). Primers were purchased from Sangon Bioengineering Co., Ltd. (Shanghai, China). Distilled water was purchased from Watson Co., Ltd. (Hong Kong, China).

### 4.2. Cell Culture and Treatment

HK-2 cells (purchased from BeNa Microbial and Cellular Resource Collection Center, Beijing) were cultured in DMEM/F-12 medium supplemented (C11330500BT, Gibco, Waltham, MA, USA) with 10% fetal bovine serum (FBS) (SA211.02, Cellmax, Beijing, China) and 1% penicillin–streptomycin (15140122, Gibco, Waltham, MA, USA) mixture at 37 °C in a 5% CO_2_ incubator.

### 4.3. Determination of Cell Viability and Cell Morphology Observation

Amino acid solutions at higher concentrations within the solubility were prepared in advance. Cysteine was prepared at a concentration of 10 mM, aspartic acid at 20 mM, tryptophan at 30 mM, and the rest of the amino acids at 50 mM. After the chiral amino acids were fully vortexed and dissolved in a complete medium, they were filtered through a 0.22 μm filter membrane to remove bacteria and set aside. A total of 100 μL of cell suspension containing 1 × 10^4^ cells was seeded in each well of a 96-well plate and incubated for 24 h. After removing the complete medium, each well was washed twice with DPBS and treated with different concentrations of 100 μL L/D-AA for 24 h [30]. Next, 10 μL of Cell Counting Kit-8 (GK10001, GLPBIO, Montclair, CA, USA) was added to each well. After 2.5 h of incubation at 37 °C, the absorbance at 450 nm was detected with a microplate reader (SPARK, TECAN, Männedorf, ZH, Switzerland). Cell viability was expressed as the ratio between the absorbance of the treatment and control groups. (All amino acids are not required except cysteine, which must be washed out of the medium before being added to CCK-8). After 15 mM L/D-AA incubation, the morphological characteristics of the cells were observed using an inverted microscope (DMi8, LEICA, Wetzlar, Germany) in the bright field with a magnification of 10× [44].

### 4.4. Detection of ROS Level

Intracellular ROS was detected by the fluorescent probe DCFH-DA using a reactive oxygen assay kit (S0033, Beyotime, Shanghai, China). A total of 1 mL of cell suspension containing 1 × 10^5^ HK-2 cells was seeded into each well of a 12-well plate and incubated for 24 h. Each well was treated with 1 mL of 10 mM L/D-AA for 24 h. Plates were removed, and positive control wells were incubated with 0.2 v% of 2 μL ROS-up for 30 min. The DCFH-DA probe was diluted to 10 µM with serum-free DMEM/F-12 medium. The plate was then taken out, and each well except the negative control was added with 0.5 mL of 10 µM DCFH-DA probe and incubated for 30 min. In the dark, cells were washed 5 times with serum-free medium to remove DCFH-DA, digested with trypsin (25200072, Gibco, Waltham, MA, USA) for 2 min, and then blown with serum-free medium. The cell pellets were suspended in DPBS after centrifugation at 1200 rpm for 5 min. Mean fluorescence intensity was measured at an excitation wavelength of 488 nm using a flow cytometer (FACSAriaIII, BD Bioscience, San Jose, CA, USA). The assay was performed in an ice bath and protected from light. Data were analyzed using the FlowJo V10 software.

### 4.5. Determination of Antioxidant Enzyme Level

A total of 2 mL of cell suspension containing 2 × 10^5^ HK-2 cells was seeded into each well of a 6-well plate and incubated for 24 h. Each well was treated with 2 mL of 10 mM L/D-AA for 24 h. Then, 200 μL of cell lysis buffer was added to lysed cells in an ice bath for 10 min. Cells were scraped with a cell scraper (3010, Corning, Corning, NY, USA) and subsequently centrifuged to collect supernatant proteins.

The protein concentration in each tube was quantified using a BCA protein assay kit (PC0020, Solarbio, Shanghai, China) by measuring the absorbance at 562 nm. The catalase activity in the cells was determined using a CAT kit (S0051, Beyotime, Shanghai, China) by measuring the absorbance at 520 nm. The MDA content in the cells was determined using an MDA kit (S0131S, Beyotime, Shanghai, China) by measuring the absorbance at 535 nm. The superoxide dismutase activity in the cells was determined using a SOD kit (WST-8 assay) (S0101S, Beyotime, Shanghai, China) by measuring the absorbance at 450 nm. The glutathione peroxidase activity in the cells was determined using a GPx kit (DTNB assay) (S0059S, Beyotime, Shanghai, China) by measuring the absorbance at 412 nm. The CAT activity, MDA content, SOD activity, and GPx activity in the samples were normalized to the protein concentrations [53]. The treated group was divided by the control group to compare the relative viability.

### 4.6. ELISA for 8-OHdG Proteins

In 60 mm dishes, cells were treated with 4 mL of 10 mM L/D-AA for 24 h. After cell counting, the cell supernatant was normalized according to the number of cells. The level of Cyt-C in the cell culture supernatant was detected according to the instructions of the 8-OHdG ELISA kit (E-EL-0028, Elabscience, Wuhan, China), and the absorbance was measured at a wavelength of 450 nm [41]. The content of the tested protein was converted according to the standard curve.

### 4.7. Cell Apoptosis Rate Detection

Cell apoptosis was tested according to Annexin V/FITC Apoptosis Detection Kit instructions (C1062, Beyotime, Shanghai, China). In 6-well plates, each well was treated with 2 mL of 10 mM, 15 mM, and 20 mM L/D-AA for 24 h. The supernatant and adherent cell pellets were collected by trypsin digestion and centrifugation. Next, 15 × 10^4^ cells were resuspended in a staining solution (containing 195 µL of binding buffer, 10 µL of PI, and 5 µL of Annexin V-FITC) and incubated for 30 min at room temperature in the dark. Finally, the mean fluorescence intensity of FITC and PI dyes was monitored by flow cytometry in the FITC and PE channels. The cells with positive Annexin V/FITC signals and negative PI signals were regarded as early apoptotic cells, while those with positive Annexin V-FITC signals and PI signals were identified as late apoptotic or necrotic cells. Data were analyzed using the FlowJo V10 software.

### 4.8. Measurement of MMP

MMP was measured by the JC-1 Apoptosis Detection Kit (40706ES60, Yeasen, Shanghai, China) [24]. In 12-well plates, cells were treated with 2 mL of 15 mM L/D-AA for 24 h. The cells were washed with DPBS, and JC-1 staining solution was added to each well and incubated at 37 °C for 20 min without light. The cells were washed twice with JC-1 staining buffer (1×). Serum-free DMEM/F-12 medium was added to each well, incubated at 37 °C for 30 min, and then washed thrice with DPBS. A total of 10 µg/mL Hoechst 33342 (C0030-50 mL, Solarbio, Beijing, China) was added to the cells, incubated at 37 °C for 15 min, and then washed thrice with DPBS. Red fluorescence (excitation at 561 nm), green fluorescence (excitation at 488 nm), and blue fluorescence (excitation at 405 nm) were observed using a confocal laser scanning microscope (DMI8, Leica, Wetzlar, Germany) at 40× magnification. Fluorescence intensity analysis was performed using Image J 1.53e. The ratio of red to green fluorescence was used to reflect the level of mitochondrial membrane potential.

### 4.9. Measurement of Intracellular pH

Intracellular pH was measured by loading cells with BCECF, AM fluorescent probe (40701ES50, Yeasen, Shanghai, China) [60]. A total of 2 μM BCECF AM staining solution was added to each well and incubated at 37 °C for 35 min without light. The cells were washed thrice with serum-free DMEM/F-12 medium. A total of 10 µg/mL Hoechst 33342 was added to the cells, incubated at 37 °C for 15 min, and then washed thrice with DPBS. Green fluorescence (excitation at 514 nm) and blue fluorescence (excitation at 405 nm) were observed using a confocal laser scanning microscope at 40× magnification. Fluorescence intensity analysis was performed using Image J 1.53e.

### 4.10. Measurement of Intracellular Free Ca^2+^ Concentration

Intracellular free Ca^2+^ concentration was measured by Rhod-2, AM fluorescent probe (440776ES50, Yeasen, Shanghai, China). A total of 4 µM Rhod staining solution was added to each well. The plate was incubated at 37 °C for 30 min in the dark. The cells were washed thrice with serum-free DMEM/F-12 medium. Serum-free DMEM/F-12 medium was added to each well, incubated at 37 °C for 30 min, and then washed thrice with DPBS. A total of 500 µL of 10 µg/mL Hoechst 33342 was added to the cells, incubated at 37 °C for 15 min, and then washed thrice with DPBS. Red fluorescence (excitation at 561 nm) and blue fluorescence (excitation at 405 nm) were observed using a confocal laser scanning microscope at 40× magnification. Fluorescence intensity analysis was performed using Image J 1.53e.

### 4.11. Measurement of Caspase-3 and Caspase-9 Protein Activities

The enzyme activities of Caspase-3 and Caspase-9 were determined according to the instructions of a Caspase-3 activity detection kit (C1116, Beyotime, Shanghai, China) and a Caspase-9 activity detection kit (C1158, Beyotime, Shanghai, China). In 60 mm dishes, cells were treated with 4 mL of 15 mM L/D-AA for 24 h. Cells were digested by trypsin. Then, the cell pellets were lysed by the cell lysis buffer in an ice bath for 15 min. The cell supernatants were collected after centrifugation, and the total protein concentration of each group was determined by the Bradford method (P0006, Beyotime, Shanghai, China). Then, 40 μL of reaction buffer, 25 μL of cell lysate, 25 μL of protein sample, and 10 μL of enzyme substrate (Ac-DEVD-pNA or Ac-LEHD-pNA) were sequentially added to each well of the 96-well plate, followed by incubation at 37 °C for 1 h. Absorbance at 405 nm was measured with a microplate reader, and pNA content in the sample was quantified according to the standard curve. The Caspase enzyme activity was normalized to the total protein concentrations [44].

### 4.12. ELISA for Bax, Bcl-2, and Cyt-C Proteins

The levels of Bax in the cytoplasm were measured by a Bax ELISA kit (ab199080, Abcam, Cambridge, MA, UK). Total cellular proteins were extracted by in situ lysis. A total of 600 μL of 1× PTR lysate was added to a 60 mm dish, and cells were lysed in an ice bath for 10 min. Cells were scraped and subsequently centrifuged to collect supernatant proteins. The total protein concentration of each group was determined using a BCA protein assay kit.

The levels of Bcl-2 in the cytoplasm were determined by a Bcl-2 ELISA kit (ab119506, Abcam, Cambridge, MA, UK). Total cellular protein was extracted by scraping, followed by lysis. Cells were scraped and centrifuged to discard the supernatant. A total of 100 μL of 1× lysis buffer was added to each well, and cells were lysed at 400 rpm for 60 min at room temperature. The supernatants were subsequently centrifuged, and protein concentrations were determined.

The levels of Cyt-C in the cytoplasm were measured by a Cyt-C ELISA kit (E-EL-H0056, Elabscience, Wuhan, China). Total cellular proteins were extracted using repeated freeze–thawing. Cells were digested with trypsin and resuspended in DPBS. Subsequently, the cell suspension was placed at −80 °C for 1 h, in liquid nitrogen for 0.5 h, and was rapidly melted by shaking in a water bath at 30 °C, which was repeated thrice. The supernatants were subsequently centrifuged to determine the protein concentration and stored in an ice bath.

The absorbance at 450 nm was detected with a microplate reader for three proteins. The tested protein contents were quantified according to the standard curve and normalized to the total protein concentrations.

### 4.13. Statistical Analysis

Data from this experiment are expressed as mean ± standard deviation (SD) of 3–6 replicate trials. GraphPad Prism 8 was used to plot the graphs and perform a multifactor analysis of variance (ANOVA) to process the data. *p* < 0.05 was considered a statistically significant difference and is indicated as error bars with *. *p* < 0.01 was considered a highly significant difference and is indicated as error bars with **. *** means *p* < 0.001; **** means *p* < 0.0001. The list of abbreviations can be found at Table S1.

## 5. Conclusions

In summary, chiral basic amino acids promoted the growth of HK-2 cells, while chiral acidic and aromatic amino acids (Glu, Asp, Trp, and Phe) inhibited cell growth. Notably, L-Glu, D-Asp, L-Trp, and D-Phe exhibited stronger inhibitory effects on cell growth compared to the other configurations. The molecular mechanism indicated that after exposure to Glu, Asp, Trp, and Phe, the imbalance between cellular ROS and antioxidant enzyme homeostasis induced oxidative stress in HK-2 cells, accompanied by DNA oxidative damage and lipid peroxidation. Additionally, mitochondrial function changes mediated cell apoptosis. This study provides reference data for defining the harmful concentrations and elucidating the mechanisms of chiral amino acids in HK-2 cells and offers a new perspective for studying cell biology from a chirality standpoint.

## Data Availability

All the data are provided within the article and Supporting Information. Data will be shared upon request (Ying Lu, The University of Chinese Academy of Sciences, luying21@mails.ucas.ac.cn).

## Supplementary Materials

The following supporting information can be downloaded at: https://www.mdpi.com/article/10.3390/ijms252413439/s1.
