# Supplementary material for: Chiral Amino Acids Mediate Mitochondria-Dependent Apoptosis of Human Proximal Tubular Epithelial Cells Under Oxidative Stress
Source: Int J Mol Sci. 2024 Dec 15;25(24):13439. doi: 10.3390/ijms252413439 (PMC11677210; doi:10.3390/ijms252413439)
Supplement: Supplementary file 1 [file ijms-25-13439-s001.zip › ijms-3347201-supplementary.pdf]

# Chiral Amino Acids Mediate Mitochondria-Dependent Apoptosis of Human Proximal Tubular Epithelial Cells under Oxidative Stress

Ying Lu [1], Yang Zhang [1], Zhaoyang Jin [1], Shuaishuai Cui [1], Li Wu [1,*] and Yujian He [1,2,*]

1 School of Chemical Sciences, University of Chinese Academy of Sciences, Beijing 100049, China
luying21@mails.ucas.ac.cn (Y.L.); jinzhaoyang20@mails.ucas.ac.cn (Z.J.);
cuishuaishuai22@mails.ucas.ac.cn (S.C.)

2 School of Future Technology, University of Chinese Academy of Sciences, Beijing 100049, China
zhangyang184@mails.ucas.ac.cn

* Correspondence: wuli@ucas.ac.cn (L.W.); heyujian@ucas.ac.cn (Y.H.)

## Supplementary File

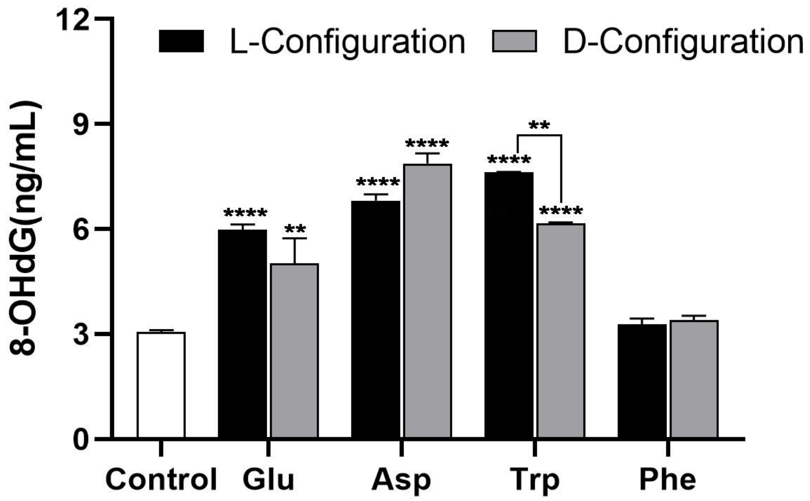


**Figure S1.** Oxidative damages of mtDNA and nuclear DNA after 10 mM of chiral amino acids acting on HK-2 cells for 24 h. (Statistical significance relative to control was marked with** $p$ < 0.01, or **** $p$ < 0.0001).

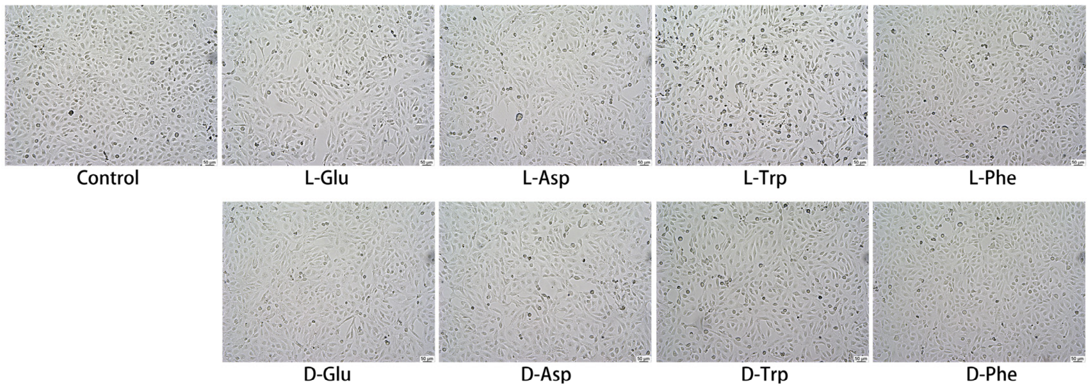


**Figure S2.** Cell morphology after 10 mM of chiral amino acids acting on HK-2 cells for 24 h, observed with an inverted microscope at a magnification of 10×. Scale bars: 50 µm.

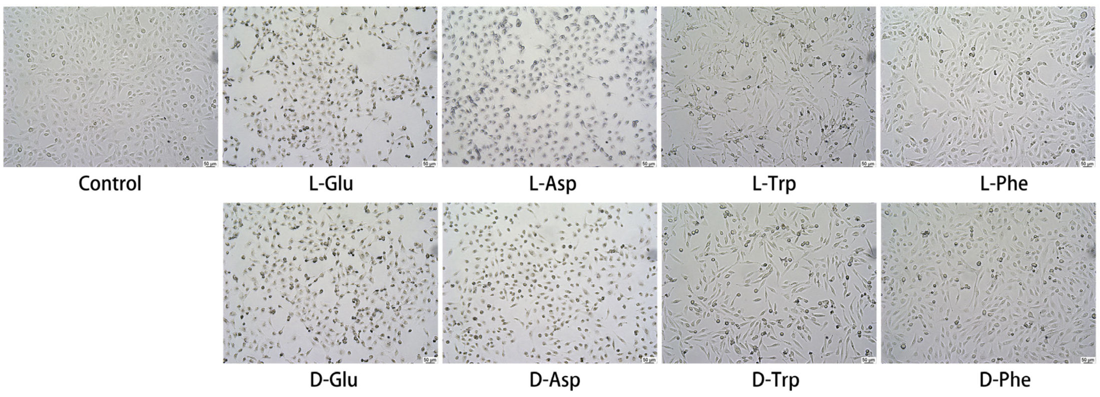


**Figure S3.** Cell morphology after 20 mM of chiral amino acids acting on HK-2 cells for 24 h, observed with an inverted microscope at a magnification of 10×. Scale bars: 50 µm.

**Table S1.** Abbreviation List

| Abbreviation | Full title |
|---|---|
| 8-OHdG | 8-hydroxy-2′-deoxyguanosine |
| Bax | Bcl-2 associated X protein |
| Bcl-2 | B-cell lymphoma-2 |
| Caspase | Cysteinyl aspartate specific proteinase |
| CAT | Catalase |
| Cyt-C | Cytochrome C |
| DAAO | D-amino acid oxidases |
| DCFH-DA | 2′,7′-dichlorodihydrofluorescein diacetate |
| DMEM/F-12 | Dulbecco's Modified Eagle Medium/Nutrient Mixture F-12 |
| DPBS | Dulbecco's Phosphate-Buffered Saline |
| FBS | Fetal bovine serum |
| GPx | Glutathione peroxidase |
| GR | Glutathione reductase |
| $H_2O_2$ | Hydrogen peroxide |
| HK-2 | Human proximal tubular epithelial cells |
| L/D-AA | L- and D- configuration amino acids |
| MDA | Malondialdehyde |
| MMP | Mitochondrial membrane potential |
| ROS | Reactive oxygen species |
| SD | Standard deviation |
| SOD | Superoxide dismutase |

Three-letter abbreviations are used for amino acids.
